# Topological properties of a self-assembled electrical network via ab initio calculation

**DOI:** 10.1038/srep41621

**Published:** 2017-02-03

**Authors:** C. Stephenson, D. Lyon, A. Hübler

**Affiliations:** 1 Department of Physics, University of Illinois at Urbana Champaign, Urbana, Illinois, USA

## Abstract

Interacting electrical conductors self-assemble to form tree like networks in the presence of applied voltages or currents. Experiments have shown that the degree distribution of the steady state networks are identical over a wide range of network sizes. In this work we develop a new model of the self-assembly process starting from the underlying physical interaction between conductors. In agreement with experimental results we find that for steady state networks, our model predicts that the fraction of endpoints is a constant of 0.252, and the fraction of branch points is 0.237. We find that our model predicts that these scaling properties also hold for the network during the approach to the steady state as well. In addition, we also reproduce the experimental distribution of nodes with a given Strahler number for all steady state networks studied.

Electrical transportation networks can be found in many disparate areas, including electrical arcs such as lightning^1^,^2^, biological information distribution systems^3^, the connections between neurons in a brain^4^, and electrical power distribution networks^5^. These type of networks are often not designed or engineered, they grow naturally in accordance to the physical laws that govern their constituents.

Complex flow networks also appear upon careful analysis of other systems. The analysis of complex time series such as EEG data reveals that understanding of the network structure of the generating process is helpful in detecting epileptic seizures^6^. Understanding of the complex network structure of the system dynamics also allows for characterization of oil-water flows^6^,^7^, and gives insight into transitions in nonlinear gas-liquid flows^8^.

Surprisingly, even though the underlying dynamics varies from system to system, certain scaling properties of the resulting networks appear to be universal for a variety of systems^9^. The scaling properties also play an important role in determining the global transportation properties of the network^10^. In this work, we consider a system that consists of many electrical conductors which self-assemble into a tree-like network in response to applied electrical voltages or currents^11^.

Experiments have shown that the degree distribution of the steady state network formed by this system is universal over a wide range of conditions and network sizes. In particular, it was found that the fraction nodes in the network with degree 1 is 25.2% of the total number of nodes, while 23.7% of nodes have degree 3 or higher^11^,^12^. It is interesting to note that this is similar to the degree distribution obtained from other growth processes, notably diffusion limited aggregation^12^ and 2D random minimal spanning trees^13^, although it is unknown why this is the case. In addition to the degree distribution, it is also known experimentally^12^ that the fractions of nodes with Strahler numbers 1, 2, and 3 is 45.5%, 27.5%, and 16.9% respectively, which is in agreement with Horton’s laws for river networks^14^,^15^. Several global optimization principles have been suggested as governing principles for the self-assembly process, notably a resistance minimizing principle^16^ and an entropy production maximization principle^17^. The mechanism by which these principles might emerge from the physical dynamics of the self-assembly process still remains unclear.

Some attempts have been made to model the self-assembly process, but these typically involve nonphysical simplifications in order to avoid the complex many-body interactions^18^,^19^,^20^. These models are unable to predict the scaling properties of the emergent networks, and predict a steady state structure which is qualitatively different from the experimentally observed structure^19^. Here we construct a model of the self-assembly process starting from fundamental electrodynamics which includes the many-body interactions by construction. We then develop a method that makes the numerical solution of the model possible. We are then able to calculate the topological properties of the emergent network starting directly from the physical laws of motion. We then use this method to calculate the degree distribution of the network as well as the distribution of nodes with a given Strahler number. This model correctly reproduces the experimentally measured results, and also predicts the topological structure of the emerging network during the formation process. Surprisingly, we find that the observed steady state degree distribution relations are also obeyed during the approach to steady state.

## Results

### Physical model

We consider the case of *i* = 1, 2, …, *N* electrically conducting spheres of radius *R*. Since the current has been experimentally measured to be quite low^21^, the conductor interactions can be modeled using electrostatics alone. In the empty regions between conductors, the electric potential 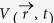 is determined by the Laplace equation


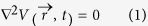


under the approximation 
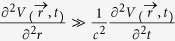
 with appropriate boundary conditions.

The total charge on conductor *i* is related to the potential via


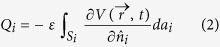


Here the integral is taken over the surface *S*_*i*_ of conductor *i* and 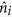 is the surface normal vector of the surface element *da*_*i*_. The permittivity of the medium is *ε*.

The conductor charges are taken to be the boundary conditions for Eq. 1 with the exception of an additional conductor *i* = 0, the ground electrode, which held at a fixed position with zero electric potential. When two or more conductors are in electrical contact, they form a new conductor with a charge equal to the sum of all the conductors in contact. If a conductor is in contact with the ground electrode, it will also have zero electric potential.

The solution of Eq. 1 with these boundary conditions allows the calculation of the electric force 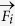 on the *i*^*th*^ conductor as another surface integral of the form


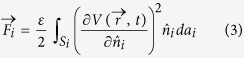


This is enough to write down the dynamics of the system:


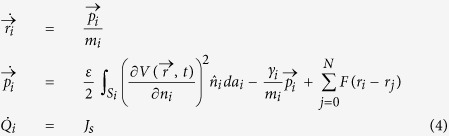


where *m*_*i*_, *γ*_*i*_ is the mass and drag coefficient of conductor *i*, respectively. A charge source term *J*_*s*_ has been added to account for the charge supplied to the system by external means. *F(r*_*i*_ − *r*_*j*_) is the contact force between conductor *i* and *j*. In this work we take the contact force between two spherical objects to be 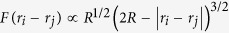 for *r*_*i*_ − *r*_*j*_ < 2 *R*, and *F(r*_*i*_ − *r*_*j*_) = 0 otherwise^22^.

### Network analysis

**Figure 1:**
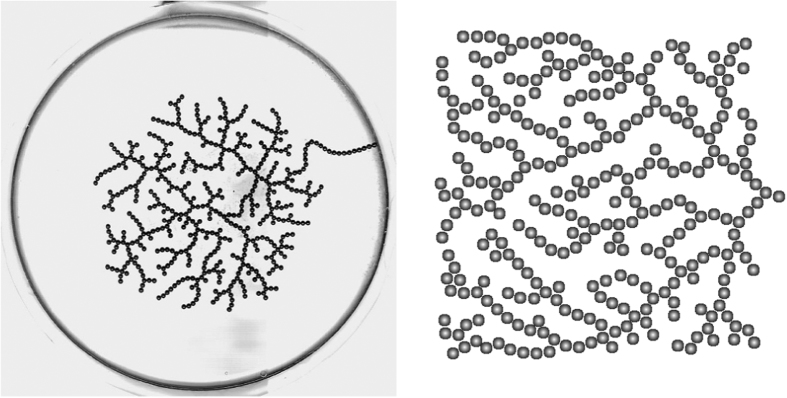
Left: Experimental steady state for *N* = 512. Right: Numerically calculated state after *t* = 120 *s* for *N* = 289.

We computed the positions of the *N* conductors in the system as a function of time for nine values of *N* between *N* = 100 and *N* = 324. These numbers were chosen to be comparable to previous experiments^11^,^12^. A comparison between the steady state produced by the experimental system and the state produced by the numerical solution of the model after *t* = 120 *s* can be seen in Fig. 1. This time was chosen such that the system reached an approximately steady state in all cases.

From the set of conductor positions obtained from the numerical solutions of the equations of motion, Eq. 4, an *N* node graph 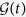 was constructed that represents the electrical network at a given time *t* in which each node corresponds to one conductor. For the analysis, two conductors *i, j* are considered to be electrically connected at time *t* if


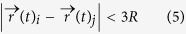


This is the connection criterion used in the experimental work^12^. The weight of the connection between nodes *i, j* is *w*_*ij*_ = 1 if the conductors are electrically connected and *w*_*i,j*_ = 0 otherwise. We then define the degree *d*_*i*_ of each node *i* to be


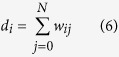


It is also possible to define an anti-arborescence 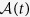 rooted at the ground electrode. To do this, each conductor *i* is assigned a direct successor *D*_*i*_ with the interpretation that the flow of charge in the network is from *i* to *D*_*i*_. The successors are then computed iteratively by defining the successor of all conductors connected to the ground electrode to be the ground electrode. In each subsequent iteration, the successor *D*_*i*_ of the *i*^*th*^ conductor is defined to be the nearest conductor that is connected to *i* and has a successor provided that *i* does not already have a successor. This process is iterated until no new successors can be assigned. Depending on the connectivity of 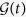, it is possible that not all of the *N* conductors will be in 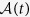 and so we will use *M* to denote the number of nodes in 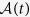.

The total number of nodes which are directly or indirectly connected to the ground electrode and have degree *d*_*i*_ = *j*, is called Δ_*j*_(*t*). This is computed as


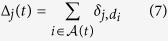


where *δ*_*i,j*_ is the Kronecker delta.

We define a branch node to be a node *i* that has a degree *d*_*i*_ ≥ 3. The total number of nodes *B(t*) which are branch nodes at time *t* can be calculated as


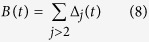


with the successors *D*_*i*_ defined, each node *i* can then be assigned a Strahler number *s*_*i*_. These are determined using the standard procedure. First, we assign *s*_*i*_ = 1 for all 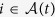 with *d*_*i*_ = 1. Then for each node that doesn’t have a Strahler number defined we set *s*_*i*_ equal to the maximum Strahler number of the nodes which have node *i* as their direct successor. If more than one node with *i* as its direct successor have the maximum Strahler number, *s*_*i*_ is incremented by *s*_*i*_ → *s*_*i*_ + 1. The number of nodes of with Strahler number *j* is *σ*_*j*_:


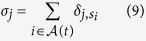


### Comparison to experimental results

**Figure 2:**
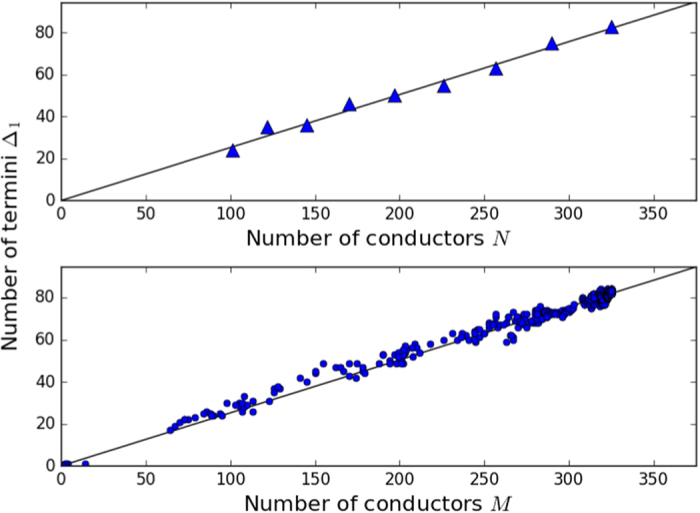
Top: Number of termini Δ_1_ as a function of total number of conductors *N* after *t* = 120 *s*. Bottom: Number of termini Δ_1_ as a function of number of conductors connected to the ground electrode *M* during the formation process out of total *N* = 324. In both plots the slope of the black line is the experimentally measured value^11^ 0.252.

Experimental results suggest a linear relationship between 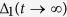 and *N*. Specifically, the relation 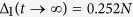 was found in steady state networks^11^. Figure 2 shows a plot of Δ_1_(*t*) vs. *N* computed from the model after *t* = 120 *s* of run time compared to experimentally observed steady state relationship.

**Figure 3:**
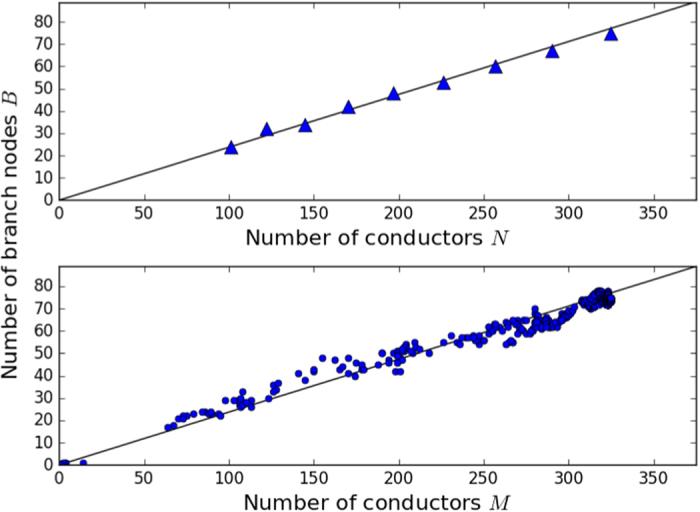
Top: Number of branch points *B* as a function of total number of conductors *N* after *t* = 120 *s*. Bottom: Number of branch nodes *B* as a function of number of conductors connected to the ground electrode *M* during the formation process out of total *N* = 324. In both plots the slope of the black line the experimentally measured value^11^ 0.237.

In addition, the relation 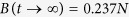 was also observed in steady state networks^11^. Figure 3 shows a plot of *B(t*) vs. *N* computed from the model after *t* = 120 *s* of run time compared to experimentally observed steady state relationship.

**Figure 4:**
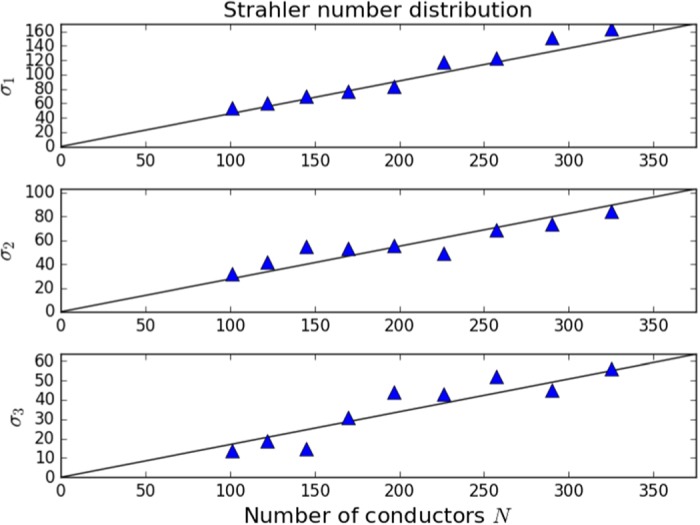
Number of nodes *σ*_*j*_ with Strahler number *j* as a function of total number of nodes. Black lines are the experimentally observed relations^12^.

A similar result was observed to hold for the distribution of nodes of a given Strahler number^12^. Experimental results show that *σ*_1_, *σ*_2_, *σ*_3_ are each linearly related to *N*. The relations are *σ*_1_ = 0.455 *N, σ*_2_ = 0.275 *N* and *σ*_3_ = 0.169 *N*. Figure 4 shows a comparison of the computed distribution of Strahler numbers as compared to the experimentally observed relations.

## Discussion

It can be seen from the model that any stationary state of the system must correspond to a connected graph. This is because any conductor that lacks a connection (either directly or through contact with other conductors) to the ground electrode will eventually experience a force directed towards the ground electrode or another conductor in contact with the ground electrode due to the accumulation of charge from the source term *J*_*s*_. Only in the event that all conductors have a connection to the ground electrode do the forces on the conductors vanish.

Experimentally, it has been noted that the stationary networks rarely have closed loops^11^,^12^. This may be due to the form of Eq. 3, which shows that the force on any surface element of a conductor is normal to the surface and directed outwards. A closed loop can be thought of a single conductor with electric field 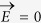 inside the loop. Any such loop may experience a force that acts to expand the loop, and thus separate the conductors that comprise it. This force can only be zero in the case that 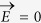 everywhere on the outside surface of the loop as well. Therefore, closed loops in the conductor network are at best neutrally stable, and unstable in the presence of any external electric field.

The stationary states of the system are thus expected to be connected acyclic graphs, or trees. For any *M* node tree 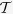, we can define the fraction *f*_*i*_(*t*) of nodes in 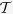 that have degree *i* as


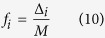


The fractions *f*_*i*_ are not completely independent. First there is a normalization constraint with maximum degree *i*_*max*_


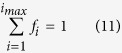


Second, there is a constraint related to the number of branches and endpoints. This can be interpreted as a statement that every branch in the tree ultimately has an end associated with it, and the number of additional branches created by a node of degree *i* is *i* − 2. This can be expressed in terms of the fractions *f*_*i*_ as


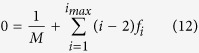


In the limit 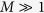, the 1/*M* term may be dropped, and equations 11 and 12 combine to give


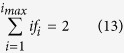


Eq. 13 implies that the average degree of nodes is 〈*i*〉 = 2 for large *M*.

For spherical particles in 2*D*, the maximum degree any node can have *i*_*max*_ = 4, as any degree larger than this would be a closed loop if connections are defined using 5. Thus there are only two independent numbers that specify the degree distribution of the network. The degree distribution can be expressed in terms of the fraction of endpoints *f*_1_ and the fraction of branch nodes *b* = *f*_3_ + *f*_4_ as follows.


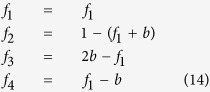


Using the experimental values of *f*_1_ = 0.252 and *b* = 0.237 we obtain the full degree distribution


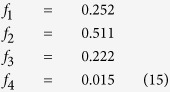


We note the apparent similarity of this degree distribution with that minimum spanning trees for random sets of nodes^13^ which have *f*_1_ = 0.253, *f*_2_ = 0.527, *f*_3_ = 0.204, and *f*_4_ = 0.016. This may suggest that the network evolves in accordance with some optimization principle, such as the resistance minimizing principle^16^, or the maximum entropy production principle^17^.

The model is able to reproduce the experimental degree distribution for self assembled electrical networks starting from the physical interaction between conductors as well as correctly explain the rarity of closed loop structures. In addition, the model predicts that the degree distribution of the network remains constant during the approach to the steady state. We are also able to reproduce the experimentally observed distribution of Strahler numbers in the network.

## Methods

### Calculation of electric potential

Solutions to Eq. 1 were generated in parallel with the red-black Gauss Seidel method^23^. The region of interest was a 2D square *L* = 5.12 *cm* on each side which was discretized into a square grid with 512 × 512 sites. Neumann boundary conditions were taken at the edges of the square region, and all sites within conductor *i* = 0 were held at zero potential. In addition, a constraint was imposed such that the total charge on each conductor with *i* > 0 remained fixed at it’s known value from Eq. 4. This constraint corresponds to finding a set of potentials {*ϕ*_*i*_} on the remaining conducting surfaces which were used as Dirichlet boundary conditions.

This is accomplished by first defining the function


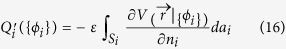


In this equation 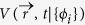 is the solution of Eq. 1 subject to the Dirichlet boundary conditions given by the set of *ϕ*_*i*_’s. Equation 16 gives the charge on the *i*^*th*^ conductor if all the conductors were held at the known fixed voltages {*ϕ*_*i*_}. With this, we construct the functions


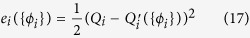


Along with the total error associated with these initial conditions


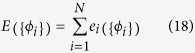


By the uniqueness property of solutions to Eq. 1, there is only one solution of the form *E*({*ϕ*_*i*_}) = 0 for Eq. 18. Therefore this solution gives the correct conductor potentials {*ϕ*_*i*_} corresponding to the charges {*Q*_*i*_}.

The problem of solving for the conductor voltages is now a root finding problem which can be solved by gradient descent. Gradient descent for this problem would involve making the update


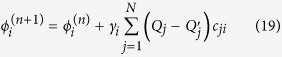


for some positive *γ*_*i*_. However, this update requires *N* sums over *N* terms, and also requires knowledge of the coefficients *c*_*ij*_. Instead, we make a local update by considering only one term of the sum


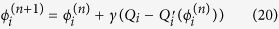


Here we have made the redefinition *γ*_*i*_*c*_*ii*_ → *γ* since *c*_*ii*_ is always positive as well^24^,^25^. This update does not require the sum over *N* terms, and does not explicitly require knowledge of *c*_*ii*_. It only requires that *γ* be chosen to ensure convergence. At each iteration, the conductor potentials are changed by an amount


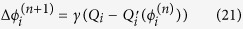


From now on we will write 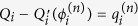. This update causes the errors *e*_*i*_ to change by


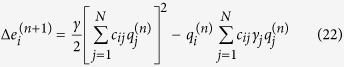


In general this can be either positive or negative. The total error *E* then changes on the (*n* + 1)^*th*^ iteration by


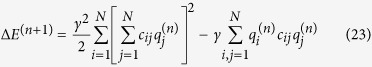


Since *c*_*ij*_ is a positive matrix^24^,^25^, the quantity 
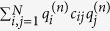
 is always positive. Therefore, the total error is always decreasing provided *γ* is chosen such that


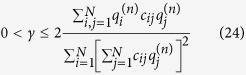


Thus the update rule in Eq. 20 converges provided *γ* is chosen to satisfy Eq. 24. In this work a value of *γ* = 7.95 *nV*/*C* was found to be sufficient for convergence. This method was iterated until a solution with *E*/*N* < 2.3 *pC* was obtained.

### Integration of the equations of motion

From the solution to Eq. 1 on the discretization grid, a bilinear interpolation was used to compute the potential between grid points. This is given by


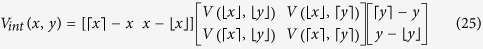


for *x*, 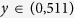. Here x is in units of grid spacing (px). This interpolation allows the computation of the electric potential in the area between grid points by approximating the behavior in the local region as changing linearly in *x* and *y* between the known values on the grid points. The surface integral in Eq. 3 was calculated from this interpolation using the trapezoid method of integration with *N*_*s*_ = 500 evenly spaced points around each conductor.


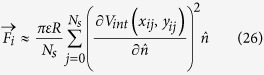


where *x*_*ij*_ = *x*_*i*_ + *R* cos (2*πj*/*N*_*s*_), and *y*_*ij*_ = *y*_*i*_ + *R* sin (2*πj*/*N*_*s*_). Here the total force vector is approximated as the vector sum of the electrical force exerted on each of the *N*_*s*_ discrete surface elements. Similarly the conductor charges from Eq. 2 are computed from


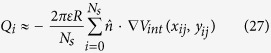


Here the charge is computed as the scalar sum of the charge on each of the *N*_*s*_ discrete surface elements.

The equations of motion in Eq. 4 were numerically integrated using Euler’s method with a timestep of Δ*t*_*m*_ = 0.01 *ms* for the mechanical degrees of freedom and Δ*t*_*e*_ = 0.001 *s* for the electrical degrees of freedom.

The mass of all conductors was set to *m*_*i*_ = 16 *g*. The fluid drag was assumed to be Stokes drag, and so *γ*_*i*_ = 6*πμR* with *μ* = 650 *cP* for castor oil^26^. The permittivity of the medium was set to be *ε* = 4.7*ε*_0_, where *ε*_0_ is the permittivity of free space. This is near the dielectric strength of castor oil^27^. For the contact dynamics we used 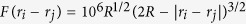. Conductors were considered to be in contact if |*r*_*i*_ − *r*_*j*_| <= 2 *R* + 2 px. The charge source was *J*_*s*_ = 3.8 *pC*/*s*.

The conductors were initially laid out in a square grid centered in the region of interest with a separation between their centers of 3 *R* and the radius of all conductors was set to be *R* = 0.8 *mm*. The ground electrode position was fixed at (*x*_0_, *y*_0_) = (496, 257). The position of the ground electrode is shifted one grid site off of the center line in the *y* direction to explicitly break symmetry.

## Additional Information

**How to cite this article**: Stephenson, C. *et al*. Topological properties of a self-assembled electrical network viaab initio calculation. *Sci. Rep.* **7**, 41621; doi: 10.1038/srep41621 (2017).

**Publisher's note:** Springer Nature remains neutral with regard to jurisdictional claims in published maps and institutional affiliations.
